# Social determinants and cardiovascular care: A focus on vulnerable populations and the Jamaica experience

**DOI:** 10.1096/fba.2020-00116

**Published:** 2021-02-02

**Authors:** Ernest Madu, Kenechukwu Mezue, Kristofer Madu

**Affiliations:** ^1^ Division of Cardiovascular Medicine Heart Institute of the Caribbean & HIC Heart Hospital Kingston Jamaica; ^2^ Division of Nuclear Cardiology Massachusetts General Hospital Harvard Medical School Boston MA USA; ^3^ School of Advanced International Studies Johns Hopkins University Washington DC USA

**Keywords:** cardiovascular disease, health inequalities, Jamaica, social determinants of health, vulnerable populations

## Abstract

The concept of social determinants of health (SDOH) describes the complex interplay of social, economic, cultural, and environmental forces that influence health and illness and result in health inequities in society. In cardiovascular disease (CVD), SDOH play a significant role in contributing to the severe morbidity and mortality that various cardiovascular diseases inflict on our societies. The components of SDOH include wealth/income, employment status, education, social interactions/support, access to medical care (including mental health services), housing, transportation, physical environment (including availability of green space, water/sanitation, air pollution, noise pollution), work environment, access to good nutrition, social and community networks, access to technology and data, exposure to crime/social disorder/violence, exposure to adverse law enforcement/bad governance, and cultural norms. Leveraging reliable SDOH data is critical to addressing healthcare needs of the community. At‐risk populations must be connected to the appropriate resources needed to overcome these barriers to access to achieve better health outcomes. This review explores this theme with a focus on several vulnerable populations and offers possible strategies to reduce these inequalities. The Heart Institute of the Caribbean (HIC) was founded in 2005 to improve access to quality medical and cardiovascular services, made available to everyone regardless of their socioeconomic status. HIC has encountered and learned to navigate a myriad structural, institutional, socio‐economic, cultural, and behavioral barriers to appropriate CVD care for vulnerable populations in Jamaica and the wider Caribbean. The successes attained and the lessons learned by HIC can be replicated in other nations to address social determinants that impede cardiovascular and medical care in vulnerable populations and may alleviate the access gap in high‐quality care in developing countries and in underserved and marginalized communities in developed countries.

## 1 INTRODUCTION

The World Health Organization (WHO) defines social determinants of health (SDOH) as “the circumstances in which people are born, grow, live, work, and age, and the systems put in place to deal with illness.”^1^ This simple but effective definition conveys the appropriate message that SDOH reflect the complex interactions of social, economic, cultural, and environmental forces that influence health and illness and result in health inequities in society. SDOH influence different communities and diseases in different ways. For example, a lack of accessible transportation in an underserved community could be a driving force behind poor patient clinic follow‐up. Poor clinic follow‐up may then result in increased complications, hospitalizations, and morbidities in congestive heart failure patients. SDOH factors, if not examined closely, may act as confounding variables impeding effective healthcare delivery. In cardiovascular disease (CVD), SDOH play a critical role in contributing to the severe morbidity and mortality that various cardiovascular diseases inflict on our societies. To improve cardiovascular healthcare outcomes for vulnerable and marginalized populations, increased attention must be given to SDOH. This review will explore this theme with a focus on several vulnerable populations and will offer possible strategies to reduce these inequities based on lessons from the Heart Institute of the Caribbean (HIC) experience.

### 1.1 Cardiovascular disease, health inequalities, and health inequities

Health inequalities are a generic term that refers to measured differences in the health status of individuals or groups. These are observations without any moral judgments and could be due to demographic, genetic, social, or other factors. Health inequities (also known as health disparities) are a subset of health inequalities that denotes an unjust difference in health status between groups that could have been avoided by reasonable means.^2^, ^3^ These health inequities are usually the result of social factors such as poor governance, corruption, or cultural exclusion.

Cardiovascular disease—mainly heart attacks and strokes—accounts for most noncommunicable Disease (NCD) deaths with approximately 17.9 million deaths yearly.^4^ An estimated 15 million people die from a NCD between the ages of 30 and 69 years; over 85% of these "premature" deaths occur in low‐ and middle‐income countries.^4^

In 1906, the Italian Political Economist, Vilfredo Pareto noted that 80% of the wealth in Italy was owned by 20% of the population, a concept that has become known as the “Pareto Principle.”^5^, ^6^, ^7^ He further opined that income and wealth distribution in all countries is uneven with a minority of the population controlling the bulk of the wealth. He suggested that all societies follow a regular pattern of income and wealth distribution which can predictably explain the inequality in the society.

The Pareto Index measures inequality of income distribution in each society. More than 100 years later, the underlying logic behind Pareto's principle and the Pareto index still exist and can largely be seen in the distribution of the social determinants of health within communities and between nations. Healthcare access and utilization appear to follow the same principle with 20% of the population utilizing 80% of global healthcare resources.^7^, ^8^ Favorable social healthcare indices are also more likely to be found in minority affluent communities in a disproportionate distribution compared to majority, poorer, or disadvantaged communities.^9^ Income inequality plagues marginalized communities and ethnic minorities. It is therefore not surprising that unfavorable social determinants of health appear to disproportionately affect ethnic minorities and other marginalized social classes.

An analysis of the technological disparities in global healthcare provides further verification of the Pareto status quo. Less developed communities do not have access to the latest health innovations and technology, as compared to their more developed counterparts. Less developed nations also lack adequate access to new advances in diagnosis and treatments due to the cost‐intensive nature of the new testing modalities and therapies, limited access to capital and lack of appropriate infrastructure and human capital. For example, during the current COVID pandemic, a study in a large health system found that Black patients were 40% less likely to participate in telemedicine compared to White patients after controlling for individual and community‐level attributes.^10^ A limited adoption of new technology and innovation by vulnerable populations is a factor contributing to health inequities.

### 1.2 Components of SDOH

The components of SDOH include wealth/income, employment status, education, social interactions/support, access to medical care (including mental health services), housing, transportation, physical environment (including availability of green space, water/sanitation, air pollution, noise pollution), work environment, access to good nutrition, social and community networks, access to technology and data, exposure to crime/social disorder/violence, exposure to adverse law enforcement/bad governance, cultural norms, access to power supply and broadband internet, good/bad governance/leadership.^1^, ^11^, ^12^ These all affect factors related to cardiovascular health and well‐being by influencing primordial and primary risk factors for cardiovascular disease, access to healthcare, compliance with therapy, and preventive medicine efforts.

Studying the individual components of SDOH is challenging as the various components interact with each other and their effects are difficult to clearly delineate. However, some components have been studied more extensively than others in respect to cardiovascular disease. For example, low educational attainment has been shown to be strongly associated with ischemic heart disease mortality and cerebrovascular disease mortality in the United States and 11 European countries.^13^ These educational disparities have also worsened with time. A study using US census data showed that a widening education‐based difference in cardiovascular death was responsible for 17.4% of the overall gap in life expectancy, second only to cancer.^9^ Education is directly related to health literacy and income, both of which have a large impact on cardiovascular disease service delivery.^14^, ^15^ Regarding employment, various job types have been shown to have associations with cardiovascular disease.^16^ Unemployment was shown to also be associated with myocardial infarction and other cardiovascular ailments.^17^, ^18^

### 1.3 Vulnerable populations

Several populations are at greater risk of poor cardiovascular healthcare in our society. We will refer to these subsets of the population as vulnerable populations. Minority ethnic populations, persons with disability, homeless people, the poor, illegal immigrants, indigenous populations, individuals in low resource nations and the LGBTQIA+population are all affected in different ways by SDOH that place barriers in their access to healthcare. Minority populations in all nations face linguistic challenges and structural barriers to limit or impede access to healthcare. Linguistic challenges also impair health literacy. In the United States for example, even though the US Department of Health and Human Services states that “healthcare providers must offer language assistance to individuals who have limited English proficiency and/or other communication needs, at no cost to them, to facilitate timely access to all healthcare and services” in its published National Standards for Culturally and Linguistically Appropriate Services in Health and Health Care, that standard is not met in most facilities and a lack of linguistic services does affect health outcomes.^19^, ^20^

In developing countries, poverty is the main factor that defines vulnerable populations.^21^, ^22^ Poverty restricts access to healthcare as poor people cannot afford to pay for both preventive and therapeutic health services and universal healthcare is not available in most developing countries. For example, in rural Jamaica, only 4% have health insurance and only 62% have access to any healthcare provider.^23^ Developing countries (compared to developed countries) also have challenges with food security/nutrition and education/literacy which are essential components of SDOH.

### 1.4 Racism, colorism, and tribalism

Bailey et al, defined structural racism as the totality of ways in which societies foster racial discrimination through mutually reinforcing discriminatory systems of housing, education, employment, earnings, benefits, credit, media, healthcare, and criminal justice.^24^ Racism is a big contributor to health disparities in various ways as it plays a role in the disparities in other relevant components of the SDOH like employment, housing, transportation, education, etc. It also influences healthcare delivery directly through inequities of access and overt or implicit provider bias.

A patient should not expect to receive a lower standard of care because of his/her race, gender, sexual orientation, age, or any other demographic characteristic. Provider bias refers to attitudes and subsequent behaviors by healthcare providers that limit patient access and choice in medical care; and these are usually informed by implicit prejudices related to the patient characteristics such as race.^25^, ^26^ Several studies have shown that provider bias is prevalent against vulnerable populations like minorities and immigrants.^27^, ^28^, ^29^ This has a direct impact in cardiovascular disease as it limits referral to specialists, referral for care, referral for testing and effective treatment. In a study that examined how physicians manage chest pain, an analysis of race‐sex interactions showed that black women were significantly less likely to be referred for catheterization than white men (odds ratio of 0.4).^30^ A systematic review found that institutional racism in the forms of residential racial segregation and mass incarceration are linked to the incidence of hypertension, and interpersonal/individual racism has a consistent direct association with increased ambulatory blood pressure.^31^ The interaction of minority populations with law enforcement agencies and the criminal justice system has also been documented to have adverse health consequences.^32^, ^33^ The recent protests in the United States calling for changes in policing and judicial sentencing for black Americans reinforce the urgency and centrality of SDOH at the societal level.^34^

In the developing world, racism takes a different form. Histories of colonial subjugation have left long‐term social and economic repercussions in former colonies across Africa, the Caribbean, and South America. Bad governance and tribalism are also recurring adverse SDOH. In ethnically homogeneous nations, overt racism is often replaced by colorism, whereby those of lighter skin tones are accorded social advantage, even in majority black populations.^35^, ^36^, ^37^ For example, Jamaica—whose population is over 90% black—suffers from deeply societally ingrained colorism.^38^, ^39^ A 2017 report conducted by the Jamaican government found that 300,000 of the nation's 2.8 million people (>10%) bleach their skin.^40^ Bleaching describes a process through which individuals apply skin lotions which often contain nephrotoxic chemicals in efforts to reduce melanin concentration and lighten skin color.^41^, ^42^ The resulting kidney damage worsens cardiovascular disease in the population, in addition to the psychosomatic stressful effects of colorism.

In other developing countries, tribalism is the bigger issue with majority ethnic groups dominating and oppressing minority groups causing similar stress effects that racism causes in these societies.^43^, ^44^ Some of this oppression has led to war and genocide, as seen in the past with the Biafran genocide (1967–1970) and the Rwandan genocide (1994) and even recently with the killings of the Rohingya Muslims in Burma (2017). In all these scenarios, a majority population preyed on a minority population that spoke a different language or had a different religion even though they were both racially indistinct.

### 1.5 Effects of stress on neurobiology and CVD

Various SDOH mediate their adverse effects on cardiovascular disease through stress‐associated neurobiological mechanisms. Poverty, unemployment, underemployment, poor work conditions, racism, segregation, poor housing, air pollution, noise pollution, exposure to crime are all adverse SDOH that lead to increased stress and anxiety. Recent research has shown that stress and anxiety lead to increased activity of the amygdala which then activates the hypothalamic–pituitary–adrenal (HPA) axis through projections to the hypothalamus, and the sympathetic nervous system (SNS) through projections to the parabrachial nucleus, nucleus tractus solitarius and the rostroventral lateral medulla.^45^, ^46^ HPA and SNS activation lead to a chronic inflammatory state and atherosclerosis and this mechanistic pathway has been shown to lead to increased adverse cardiovascular events in cohort studies.^47^, ^48^, ^49^

## 2 THE HEART INSTITUTE OF THE CARIBBEAN PARADIGM

The Heart Institute of the Caribbean (HIC) was founded in 2005 with the conviction that access to quality medical and cardiovascular services should be available to everyone in the Caribbean regardless of their socioeconomic status. This mission is anchored on the belief that, at‐risk populations in all nations and communities (both affluent and poor) must be connected to appropriate interventions needed to overcome the barriers to access and achieve better health outcomes.^50^

Cardiovascular disease is the number one cause of death, hospitalization and disability in the West Indies.^4^ Jamaica is one of the largest Caribbean islands, with a population of almost three million people, but lacked adequate number of healthcare professionals, with <3000 doctors available.^51^ In response, HIC was established to address the dearth of high‐quality cardiovascular care on the island.^52^

**FIGURE 1:**
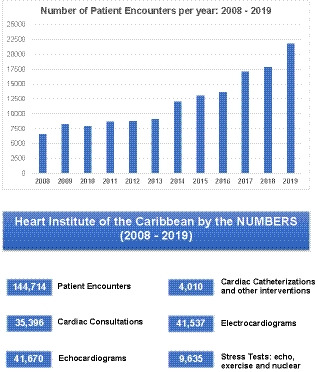
HIC through the last decade

The HIC Heart Hospital in Kingston is the only dedicated full‐service tertiary cardiac center in the English‐speaking Caribbean region that provides full outpatient and inpatient services including complex cardiac surgery, percutaneous coronary intervention, peripheral interventions, electrophysiology procedures and nuclear imaging under one roof. The Kingston facility features an ultra‐modern operating theatre suite, two interventional cardiology suites, a cardiac emergency room, an 11‐bed cardiac ICU, and a telemetry unit, with an emergency cardiac ambulance service available 24 h a day. There are also additional clinics and satellite locations in other towns on the island (Mandeville, established in 2007, Ocho Rios in 2010, Spanish Town in 2015 and Montego Bay (with an additional Interventional Cardiology suite) in 2020) which provide outpatient care and diagnostics. The arrival of HIC’s facilities greatly improved access for the population. The patient growth and demand over the 15‐year period has been substantial (see Figure 1).

Before the establishment of HIC, there was no cardiac center of excellence in Jamaica or anywhere in the English‐speaking Caribbean resulting in severe limitation of access to CVD care for the most vulnerable. The more affluent members of the society found care mostly in the United States with medical flights to Miami and other cities in Florida as the only option. The establishment of HIC has reduced the need for these flights as excellent high‐quality cardiovascular services are now offered on the island.

Bureaucratic hurdles were a significant challenge in the initial efforts to establish the HIC. A disjointed and unwieldy approval process added significantly to the financial risk exposure and general cost of doing business. This is common with developing countries and arises partly because of skepticism and partly because of the complexity of the operations that HIC was proposing. Surmounting these hurdles required patience, advocacy and developing relationships with local stakeholders with the aim of addressing the unmet needs in a way that aligned with the values of the community. There was a strong effort made among local stakeholders to change the paradigm from short‐term fixes and a dependence on foreign aid to long‐term meaningful and sustainable development.

## 3 POSSIBLE SOLUTIONS TO SDOH INEQUITIES

Addressing SDOH directly will need a multipronged, long‐term strategy. It will involve engagement of all stakeholders, public–private partnerships and multilateral agencies with defined targets that will aim at reducing health inequalities in various facets of the society and the economy. Interventions should include a combination of programs both at the population level to shift the distribution of cardiovascular risk and also at the individual level focusing on high risk members of society.^11^ Interventions should include civic and political engagement, and vectors of change should include schools, places of worship, government agencies, socio‐cultural groups, government policy, non‐governmental organizations, and other stakeholders.

One important agent that can be employed is technology. Mobile technology particularly has the potential to revolutionize access to care and improvement in other SDOH especially in its ability to create information parity in the population.^53^, ^54^ Mobile health technology can be used to reduce disparities in cardiovascular disease outcomes by overcoming such barriers as limited access to providers, difficulty communicating with providers, and inadequate communication between patients and providers regarding symptoms.^55^ For example, HIC has leveraged telemedicine extensively in the organization by employing a robust telemedicine platform (in partnership with Netmedical New Mexico, USA) that connects patients in rural locations in Jamaica and other Caribbean Islands via video to specialists at HIC and improves access to skill and expertise which ultimately benefits the population.

A common hindrance to effective solutions is short political cycles. Most interventions will need governmental support or oversight and the short political cycle hinders long‐term planning to address these SDOH possible. Funding from government agencies and philanthropists for the interventions has dropped significantly in the current coronavirus disease (COVID‐19) pandemic.

### 3.1 HIC’s models to address the high cost of care

The cost of high‐quality cardiovascular care in a low resource country is daunting. The Jamaican population is largely uninsured/underinsured with only approximately 15% of the population having health insurance.^23^ Individuals mostly finance their healthcare needs out‐of‐pocket and this is a huge burden to most patients.

A cross‐subsidy model is utilized at HIC to provide access to HIC services to all Jamaicans irrespective of socioeconomic status. This model is executed through a differential pricing model where a standard rate is offered to patients who have health insurance or a third‐party guarantor (such as a corporate sponsor), and a markedly subsidized rate is offered to patients who are paying solely out of pocket or are indigent.

The HIC Cares Membership program is a subscription scheme designed to improve access in a cost‐effective manner. The program gives participants unlimited free cardiology consultations, electrocardiograms, and significant discounts on most HIC services for a nominal yearly fee of only USD$275 (<$1 per day). The program currently has about 3000 recurrent members and is currently being expanded through a partnership with a major insurance broker.

Existing banks and financial institutions in Jamaica are reluctant to lend money to patients for healthcare needs especially without collateral requirements. The HIC has partnered with a financial company to establish a micro‐credit company—the first and only micro‐credit lender in Jamaica—with the aim of providing unsecured loans to patients to access health services. The HIC has also partnered with the Caribbean community of retired persons (CCRP) to create specific products at a subsidized rate for their 10,000 members. These partnerships increase access to healthcare for low‐income people who otherwise would not have been able to pay for it.

The HIC Foundation was established in 2008 with a focus to financing care for the most vulnerable patients. In the past decade, it has provided about US$1.5 million annually in free or subsidized care to indigent patients. The foundation is primarily internally funded by a percentage of proceeds from HIC and donations are also welcome from the public. The foundation also implements and supports free cardiac screening programs, sponsors youth development programs and sporting activities and funds free cardiac care for patients who are unable to pay for their own care.

For these innovations in access to care, the HIC has been given several accolades including the “Nation Builder Award” and the “Innovation Award” from the National Commercial Bank in Jamaica in October 2014 and the “Corporate Social Responsibility Award” 2 years in a row from the American Chamber of Commerce in Jamaica in October 2015 and October 2016.

### 3.2 HIC—technology, innovation, and education

HIC recognized that appropriate use of technology is fundamental for sustainable healthcare development.^8^ The program at HIC was therefore designed to make maximum use of the advances in technology focusing on multi‐modality systems that are readily adaptable to low resource settings, easily serviceable, and durable enough to withstand the stresses of the local environment. This enabled HIC to provide high‐quality cardiovascular technology at a fraction of the cost obtainable in the developed Western countries.

HIC has developed “Practice Partners” in all 14 parishes across Jamaica. The Practice Partner program builds a relationship between the HIC and general practitioners in the community with HIC assisting them in providing cardiology consultation and diagnostic services. In this program, electrocardiograms, echocardiograms, ambulatory blood pressure monitoring and Holter monitoring are carried out on‐site at the practice partner location, with HIC providing support and remote interpretation. For example, the HIC mobile echocardiography unit provides echocardiograms at satellite locations and practice partner locations with the images sent to a cloud‐based archiving and retrieval system provided by SIMMS Canada with capabilities for real‐time, rapid interpretation by a HIC cardiologist working remotely. This reduces the inequities of access to healthcare in the general population which directly tackles a major SDOH. We are currently expanding the Practice Partner program to other Islands in the region to bring high‐quality cardiovascular care closer to the population.

HIC focuses on local skills transfer and acquisition and the training of local personnel to improve internal capacity and to minimize the need for dependence on foreign entities. In the past 15 years, HIC has trained echocardiographers, nuclear technologists, Cath laboratory technicians, Pacemaker Technicians, and other cardiac ancillary staff and has provided mentorship for healthcare trainees in basic cardiovascular medicine care and diagnostics. HIC also works closely with other local and regional institutions and facilities to offer physicians at various career stages opportunities for cardiology rotations with HIC specialists. HIC has also developed strong collaborations with international centers of excellence in cardiovascular care including such centers as University of Pennsylvania Perelman School of Medicine and Yale University. A monthly telecardiology conference is held with HIC and Penn faculty focused on carefully selected topics addressing the quality of local care in a case‐based discussion format. This conference is free for local doctors, nurses, and technicians with continuing medical education (CME) points awarded by the Medical Council of Jamaica and the University of Pennsylvania. These collaborations have greatly enhanced access to HIC patients and significantly improved the quality of care for patients in the region.

Research is a major component of the HIC value proposition. Over the past 15 years, HIC investigators have made significant contributions in the scientific literature documenting cardiovascular diseases and therapeutic interventions in the Caribbean.

HIC has encountered and has learned to navigate a myriad structural, institutional, socio‐economic, cultural, and behavioral barriers that have acted as adverse social determinants to appropriate CVD care for vulnerable populations in Jamaica and the wider Caribbean. These same challenges that HIC met in Jamaica are present in various forms in other low resource nations around the world and within poorer and minority communities in the more affluent nations. The HIC experience can certainly be replicated in other nations to address social determinants that impede cardiovascular and medical care in vulnerable populations, and in so doing, help build sustainable healthcare capacity and alleviate the access gap in high‐quality care in other regions. Developed countries can also draw on these lessons and apply strategies to address inequities and access to care issues in vulnerable populations within their borders.

## 4 CONCLUSION

This review has explored SDOH—the complex interactions of social, economic, cultural, and environmental forces—in the context of cardiovascular disease care, particularly among vulnerable populations. The review also examined the experience of HIC in Jamaica. The HIC experience from the past 15 years demonstrates that high‐quality cardiovascular care anchored on smart use of technology and expertise is achievable in low resource nations of the world if driven by the appropriate dose of passion and commitment.

## CONFLICT OF INTEREST

The authors declare that they have no conflict of interest.

## AUTHOR CONTRIBUTIONS

E. Madu, K. Mezue, and K. Madu wrote the paper.

## References

[1] Marmot M , Friel S , Bell R , Houweling TAJ , Taylor S . Commission on social determinants of health. Closing the gap in a generation: health equity through action on the social determinants of health. Lancet. 2008;372(9650):1661‐1669. 10.1016/S0140-6736(08)61690-6 18994664

[2] Arcaya MC , Arcaya AL , Subramanian SV . Inequalities in health: definitions, concepts, and theories. Glob Health Action. 2015;8(1):27106. 10.3402/gha.v8.27106 26112142 PMC4481045

[3] Pickett KE , Wilkinson RG . Immorality of inaction on inequality. BMJ. 2017;356:j556. 10.1136/bmj.j556 28179272

[4] GBD 2015 Risk Factors Collaborators . Global, regional, and national comparative risk assessment of 79 behavioural, environmental and occupational, and metabolic risks or clusters of risks, 1990–2015: a systematic analysis for the Global Burden of Disease Study 2015. Lancet. 2016;388(10053):1659‐1724. 10.1016/S0140-6736(16)31679-8 27733284 PMC5388856

[5] Leo Y , Fleury E , Alvarez‐Hamelin JI , Sarraute C , Karsai M . Socioeconomic correlations and stratification in social‐communication networks. J R Soc Interface. 2016;13(125):20160598 10.1098/rsif.2016.0598 27974571 PMC5221523

[6] Wang J , Fu F , Wang L . Effects of heterogeneous wealth distribution on public cooperation with collective risk. Phys Rev E Stat Nonlin Soft Matter Phys. 2010;82(1 Pt 2):016102. 10.1103/PhysRevE.82.016102 20866684

[7] Gibbard A . Health care and the prospective Pareto principle. Ethics. 1984;94(2):261‐282. 10.1086/292532 11651754

[8] Ernest M . World Class Health Care. 2007. https://www.ted.com/talks/ernest_madu_world_class_health_care

[9] Meara ER , Richards S , Cutler DM . The gap gets bigger: changes in mortality and life expectancy, by education, 1981–2000. Health Aff (Millwood). 2008;27(2):350‐360. 10.1377/hlthaff.27.2.350 18332489 PMC2366041

[10] Chunara R , Zhao Y , Chen J , et al. Telemedicine and Healthcare Disparities: A cohort study in a large healthcare system in New York City during COVID‐19. J Am Med Inform Assoc. 2020; 10.1093/jamia/ocaa217 PMC7499631 32866264

[11] Havranek EP , Mujahid MS , Barr DA , et al. Social determinants of risk and outcomes for cardiovascular disease: A scientific statement from the American Heart Association. Circulation. 2015;132(9):873‐898. 10.1161/CIR.0000000000000228 26240271

[12] Reshetnyak E , Ntamatungiro M , Pinheiro LC , et al. Impact of multiple social determinants of health on incident stroke. Stroke. 2020;51(8):2445‐2453. 10.1161/STROKEAHA.120.028530 32673521 PMC9264323

[13] Mackenbach JP , Cavelaars AE , Kunst AE , Groenhof F . Socioeconomic inequalities in cardiovascular disease mortality; an international study. Eur Heart J. 2000;21(14):1141‐1151. 10.1053/euhj.1999.1990 10924297

[14] Evangelista LS , Rasmusson KD , Laramee AS , et al. Health literacy and the patient with heart failure–implications for patient care and research: a consensus statement of the Heart Failure Society of America. J Card Fail. 2010;16(1):9‐16. 10.1016/j.cardfail.2009.10.026 20123313 PMC2909843

[15] Berkman ND , Sheridan SL , Donahue KE , Halpern DJ , Crotty K . Low health literacy and health outcomes: an updated systematic review. Ann Intern Med. 2011;155(2):97‐107. 10.7326/0003-4819-155-2-201107190-00005 21768583

[16] Davila EP , Kuklina EV , Valderrama AL , Yoon PW , Rolle I , Nsubuga P . Prevalence, management, and control of hypertension among US workers: does occupation matter? J Occup Environ Med. 2012;54(9):1150‐1156. 10.1097/JOM.0b013e318256f675 22885710

[17] Dupre ME , George LK , Liu G , Peterson ED . The cumulative effect of unemployment on risks for acute myocardial infarction. Arch Intern Med. 2012;172(22):1731‐1737. 10.1001/2013.jamainternmed.447 23401888

[18] Kivimäki M , Vahtera J , Pentti J , Ferrie JE . Factors underlying the effect of organisational downsizing on health of employees: longitudinal cohort study. BMJ. 2000;320(7240):971‐975. 10.1136/bmj.320.7240.971 10753148 PMC27336

[19] Culturally and Linguistically Appropriate Services . Think Cultural Health. https://thinkculturalhealth.hhs.gov/. Accessed August 31, 2020.

[20] Blendon RJ , Buhr T , Cassidy EF , et al. Disparities in health: perspectives of a multi‐ethnic, multi‐racial America. Health Aff (Millwood). 2007;26(5):1437‐1447. 10.1377/hlthaff.26.5.1437 17848456

[21] Peters DH , Garg A , Bloom G , Walker DG , Brieger WR , Rahman MH . Poverty and access to health care in developing countries. Ann N Y Acad Sci. 2008;1136:161‐171. 10.1196/annals.1425.011 17954679

[22] Orach CG . Health equity: challenges in low income countries. Afr Health Sci. 2009;9(Suppl 2):S49‐S51. 20589106 PMC2877288

[23] Bourne PA . Social determinants of self‐evaluated good health status of rural men in Jamaica. Rural Remote Health. 2009;9(4):1280. 19961261

[24] Bailey ZD , Krieger N , Agénor M , Graves J , Linos N , Bassett MT . Structural racism and health inequities in the USA: evidence and interventions. Lancet. 2017;389(10077):1453‐1463. 10.1016/S0140-6736(17)30569-X 28402827

[25] Solo J , Festin M . Provider bias in family planning services: a review of its meaning and manifestations. Glob Health Sci Pract. 2019;7(3):371‐385. 10.9745/GHSP-D-19-00130 31515240 PMC6816811

[26] FitzGerald C , Hurst S . Implicit bias in healthcare professionals: a systematic review. BMC Med Ethics. 2017;18: 10.1186/s12910-017-0179-8 PMC5333436 28249596

[27] Blair IV , Havranek EP , Price DW , et al. Assessment of biases against Latinos and African Americans among primary care providers and community members. Am J Public Health. 2013;103(1):92‐98. 10.2105/AJPH.2012.300812 23153155 PMC3518332

[28] Blair IV , Steiner JF , Havranek EP . Unconscious (implicit) bias and health disparities: where do we go from here? Perm J. 2011;15(2):71‐78. 10.7812/tpp/11.979 PMC3140753 21841929

[29] Sabin J , Nosek BA , Greenwald A , Rivara FP . Physicians’ implicit and explicit attitudes about race by MD race, ethnicity, and gender. J Health Care Poor Underserved. 2009;20(3):896‐913. 10.1353/hpu.0.0185 19648715 PMC3320738

[30] Schulman KA , Berlin JA , Harless W , et al. The effect of race and sex on physicians’ recommendations for cardiac catheterization. N Engl J Med. 1999;340(8):618‐626. 10.1056/NEJM199902253400806 10029647

[31] Brondolo E , Love EE , Pencille M , Schoenthaler A , Ogedegbe G . Racism and hypertension: a review of the empirical evidence and implications for clinical practice. Am J Hypertens. 2011;24(5):518‐529. 10.1038/ajh.2011.9 21331054

[32] McLeod MN , Heller D , Manze MG , Echeverria SE . Police interactions and the mental health of Black Americans: a systematic review. J Racial Ethn Health Disparities. 2020;7(1):10‐27. 10.1007/s40615-019-00629-1 31482464

[33] Bor J , Venkataramani AS , Williams DR , Tsai AC . Police killings and their spillover effects on the mental health of black Americans: a population‐based, quasi‐experimental study. Lancet. 2018;392(10144):302‐310. 10.1016/S0140-6736(18)31130-9 29937193 PMC6376989

[34] Krieger N . ENOUGH: COVID‐19, structural racism, police brutality, plutocracy, climate change‐and time for health justice, democratic governance, and an equitable, sustainable future. Am J Public Health. 2020;110(11):1620‐1623. 10.2105/AJPH.2020.305886 32816556 PMC7542259

[35] Laidley T , Domingue B , Sinsub P , Harris KM , Conley D . New evidence of skin color bias and health outcomes using sibling difference models: a research note. Demography. 2019;56(2):753‐762. 10.1007/s13524-018-0756-6 30627966 PMC6449491

[36] Oh H , Jacob L , Anglin DM , Koyanagi A . Perceived skin tone discrimination and psychotic experiences among Black Americans: Findings from the National Survey of American Life. Schizophr Res. 2020. 10.1016/j.schres.2020.11.033 33234422

[37] Slaughter‐Acey JC , Brown TN , Keith VM , Dailey R , Misra DP . A tale of two generations: Maternal skin color and adverse birth outcomes in Black/African American women. Soc Sci Med. 2020;265:113552. 10.1016/j.socscimed.2020.113552 33277068 PMC7781157

[38] Benn EKT , Deshpande R , Dotson‐Newman O , et al. Skin bleaching among African and Afro‐Caribbean women in New York City: primary findings from a P30 Pilot Study. Dermatol Ther (Heidelb). 2019;9(2):355‐367. 10.1007/s13555-019-0297-y 31020513 PMC6522580

[39] Charles CAD , McLean S‐K . Body image disturbance and skin bleaching. Br J Psychol. 2017;108(4):783‐796. 10.1111/bjop.12241 28233898

[40] Chappell K . Many Jamaicans bleach their skin, but few people talk about it. A dancehall star wants to change that. Washington Post. https://www.washingtonpost.com/world/2018/10/31/jamaican‐dancehall‐star‐takes‐her‐countrys‐discrimination‐against‐dark‐skin/. Accessed December 12, 2020.

[41] Ricketts P , Knight C , Gordon A , Boischio A , Voutchkov M . Mercury exposure associated with use of skin lightening products in Jamaica. J Health Pollut. 2020;10(26):200601. 10.5696/2156-9614-10.26.200601 32509402 PMC7269324

[42] Mahé A , Ly F , Perret J‐L . Systemic complications of the cosmetic use of skin‐bleaching products. Int J Dermatol. 2005;44(Suppl 1):37‐38. 10.1111/j.1365-4632.2005.02810.x 16187958

[43] Friedman RA . Overcoming Tribalism. Psychiatr Serv. 2018;69(9):946‐947. 10.1176/appi.ps.201800259 30045662

[44] Dixon AR . Colorism and classism confounded: perceptions of discrimination in Latin America. Soc Sci Res. 2019;79:32‐55. 10.1016/j.ssresearch.2018.12.019 30857667

[45] Gilpin NW , Herman MA , Roberto M . The central amygdala as an integrative hub for anxiety and alcohol use disorders. Biol Psychiatry. 2015;77(10):859‐869. 10.1016/j.biopsych.2014.09.008 25433901 PMC4398579

[46] Zhang X , Ge TT , Yin G , Cui R , Zhao G , Yang W . Stress‐induced functional alterations in amygdala: implications for neuropsychiatric diseases. Front Neurosci. 2018;12: 367–375. 10.3389/fnins.2018.00367 29896088 PMC5987037

[47] Ulrich‐Lai YM , Herman JP . Neural regulation of endocrine and autonomic stress responses. Nat Rev Neurosci. 2009;10(6):397‐409. 10.1038/nrn2647 19469025 PMC4240627

[48] Tawakol A , Ishai A , Takx RAP , et al. Relation between resting amygdalar activity and cardiovascular events: a longitudinal and cohort study. Lancet. 2017;389(10071):834‐845. 10.1016/S0140-6736(16)31714-7 28088338 PMC7864285

[49] Fiechter M , Roggo A , Burger IA , et al. Association between resting amygdalar activity and abnormal cardiac function in women and men: a retrospective cohort study. Eur Heart J Cardiovasc Imaging. 2019;20(6):625‐632. 10.1093/ehjci/jez047 31083711

[50] Madu EC , Tulloch‐Reid E , Edwards P , Baugh DS , Kong BW . Developing sustainable cardiovascular care for low‐resource nations. J Am Coll Cardiol. 2009;54(11):1038‐1039. 10.1016/j.jacc.2008.07.077 19729124

[51] Knight‐Madden J , Gray R . The accuracy of the Jamaican national physician register: a study of the status of physicians registered and their countries of training. BMC Health Serv Res. 2008;8(1):253. 10.1186/1472-6963-8-253 19077244 PMC2614992

[52] Denbow CE . A history of cardiology in Jamaica. West Indian Med J. 2004;53(3):184‐187. 15352749

[53] Zhang X , Hailu B , Tabor DC , et al. Role of Health Information Technology in Addressing Health Disparities: Patient, Clinician, and System Perspectives. Med Care. 2019;2:S115‐S120. 10.1097/MLR.0000000000001092 PMC6589829 31095049

[54] van Veen T , Binz S , Muminovic M , et al. Potential of mobile health technology to reduce health disparities in underserved communities. West J Emerg Med. 2019;20(5):799‐802. 10.5811/westjem.2019.6.41911 31539337 PMC6754190

[55] Gonzalez BD . Promise of mobile health technology to reduce disparities in patients with cancer and survivors. JCO Clin Cancer Inform. 2018;2:1‐9. 10.1200/CCI.17.00141 PMC6338426 30652578

